# Radiotherapy With Hydrogen Peroxide-Soaked Gauze for Preauricular Sebaceous Carcinoma

**DOI:** 10.7759/cureus.27464

**Published:** 2022-07-29

**Authors:** Akiko Adachi, Takahiro Oike, Masaaki Tamura, Norichika Ota, Tatsuya Ohno

**Affiliations:** 1 Department of Radiation Oncology, Gunma University Graduate School of Medicine, Maebashi, JPN; 2 Heavy Ion Medical Center, Gunma University, Maebashi, JPN; 3 Department of Dermatology, Sano Kosei General Hospital, Sano, JPN

**Keywords:** radiotherapy, extremely high age, hydrogen peroxide, extraocular, sebaceous carcinoma

## Abstract

Sebaceous carcinoma is a rare and aggressive malignant tumor deriving from the adnexal epithelium of the sebaceous glands. The case of inoperable preauricular sebaceous carcinoma treated with definitive radiotherapy is first reported herein. Radiotherapy of 60 Gy in 30 fractions was combined with a hydrogen peroxide-soaked gauze bolus aiming at potential radiosensitization. Macroscopic complete remission was achieved eight months after radiotherapy with tolerable adverse effects. Although further clinical studies are needed, radiotherapy with a hydrogen peroxide-soaked gauze bolus can be an effective and tolerable treatment for inoperable patients with extraocular sebaceous carcinoma.

## Introduction

Sebaceous carcinoma is a rare and aggressive malignant tumor derived from the adnexal epithelium of the sebaceous glands [1]. Sebaceous carcinoma commonly occurs in adults >60 years old, primarily in the eyelids and followed by the head and neck as well as the body trunk [2]. The survival rate for sebaceous carcinoma is 92% and 86% at 5 and 10 years, respectively [3]. Although surgery remains the mainstay of definitive treatment for sebaceous carcinoma, radiotherapy may be an option for those who are considered inoperable or reject surgery [2,3]. However, the optimal radiation dose for controlling sebaceous carcinoma remains unclear because of its rarity. Previous reports indicate that the tumors arising from the eyelid require at least 55 Gy, whereas data are lacking regarding tumors located at the extraocular lesions [3].

In recent years, hydrogen peroxide (H_2_O_2_) has attracted interest as a radiosensitizer [4-6]. For superficial tumors, H_2_O_2_-soaked gauze can be used as a bolus for radiotherapy [6-8]. Currently, several case reports have demonstrated that the combination of radiotherapy with an H_2_O_2_-soaked gauze bolus is effective and tolerable [6-8].

Here, a rare case of inoperable extraocular (i.e., preauricular) sebaceous carcinoma treated with definitive radiotherapy is reported. Since the control probability for this malignancy at the radiation dose conventionally used for common skin cancers (i.e., 60-66 Gy) remained unclear, an H_2_O_2_-soaked gauze bolus was used aiming at potential radiosensitization [3].

## Case presentation

**Figure 1:**
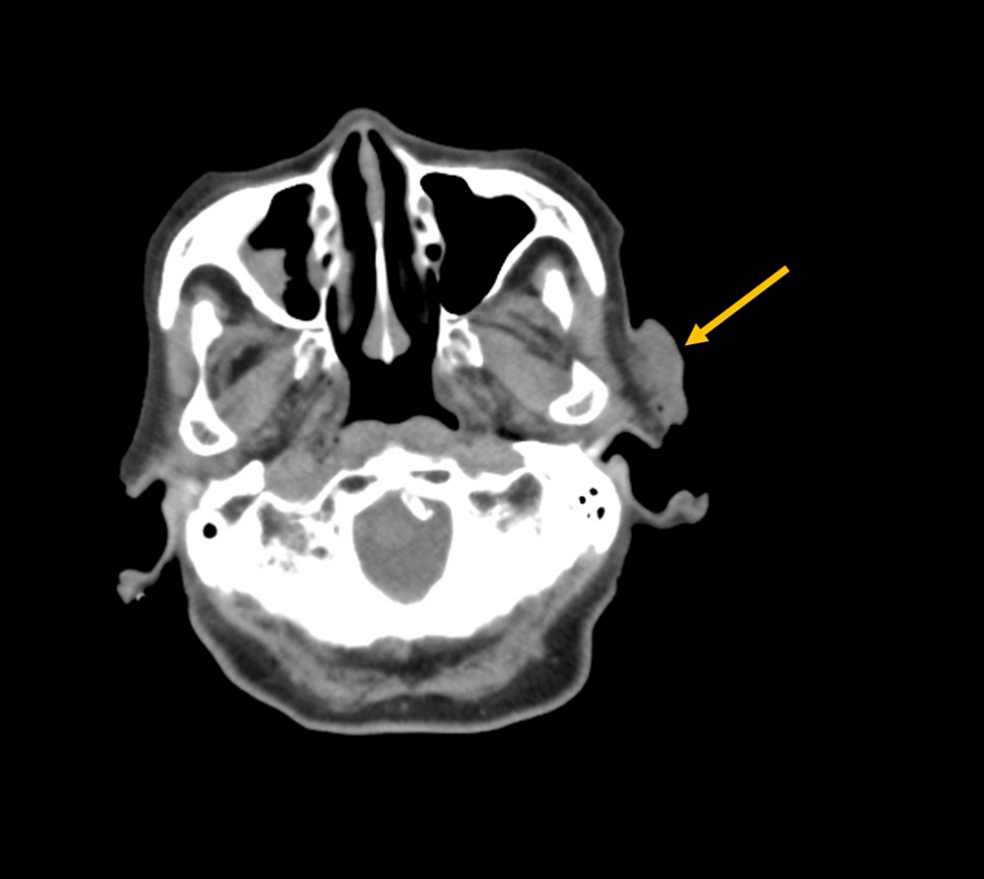
Computed tomography image obtained before radiotherapy. The arrow shows a tumor.

A 97-year-old woman was referred to the radiation oncology unit from the dermatology unit of the hospital of the current study for the treatment of preauricular sebaceous carcinoma. The patient had been living in a nursing home and experiencing exudation, bleeding, and pain from the tumor for one month before the referral, resulting in impaired quality of life (QOL). The skin tumor was 3.5 cm in diameter and was diagnosed clinically as stage II (T2N0M0) following the eighth edition of the Union for International Cancer Control (Figure 1).

The tumor was diagnosed pathologically as sebaceous carcinoma based on the positivity of the epithelial membrane antigen, androgen receptor, and factor XIIIa [2,3]. Definitive radiotherapy was performed because the patient was considered inoperable due to extremely high age.

**Figure 2:**
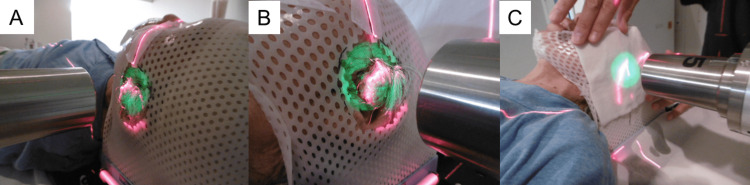
Radiotherapy planning and actual treatment. (A, B) According to tumor size, a 6- (50 Gy) and 5- (10 Gy) cm-diameter cone (for the former and the latter, respectively) was set to cover the clinical target volume (i.e., the gross tumor plus margin of at least a 1 cm). (C) Application of the hydrogen peroxidase-soaked gauze during the radiotherapy session.

Using 6-MeV electrons, 60 Gy was delivered in 30 fractions at five fractions per week (Figure 2). The source-skin distance was 100 cm. The clinical target volume (CTV) was defined as the gross tumor with a margin of at least 1 cm around the tumor. To cover the CTV, a 6-cm-diameter cone was used for the former 50 Gy, followed by a 5-cm-diameter cone for the latter 10 Gy to accommodate tumor shrinkage. An H_2_O_2_-soaked gauze (5 mm thick) was freshly prepared and placed on the tumor surface at each radiotherapy session.

Practically, when the patient entered the treatment room, a nurse poured Oxydol (i.e., 2.5-3.5 w/v% H_2_O_2_; KENEI Pharmaceutical Co. Ltd., Osaka, Japan) into a plastic bag, and gauze was soaked completely in the bag. The tumor showed a fair shrinkage as the treatment progressed.

**Figure 3:**
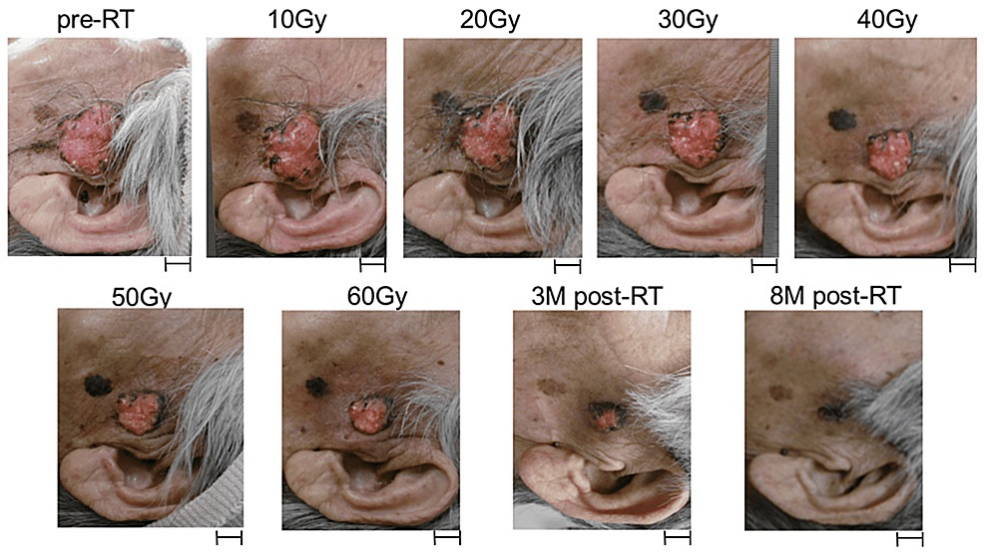
Representative pictures showing tumor shrinkage over the course of treatment. Scales 1 cm, M months, and RT radiotherapy.

Pain and exudation and bleeding from the tumor resolved upon treatment completion. The tumor showed further remission three months after radiotherapy, and macroscopic complete remission was achieved eight months after radiotherapy, which was the last observation date. The patient experienced grade 2 radiation dermatitis based on the Common Terminology Criteria for Adverse Events 5.0, which resolved at two months post-radiotherapy without topical medication and skincare. The patient is currently enjoying life in a nursing home while maintaining QOL (Figure 3).

## Discussion

Sebaceous carcinoma is a rare malignancy, and almost 70% of sebaceous carcinoma arises on the eyelids. Although no currently standardized treatment for sebaceous carcinoma exists, surgical resection has been considered the first choice. Disease-related mortality in surgically treated patients is reported to range from 6% to 30% [9,10]. Radiotherapy can be an alternative option for inoperable cases because of poor general condition, cosmetic concerns, or the patient’s refusal, and a few studies have reported the outcome of definitive radiotherapy for sebaceous carcinoma. Hata et al. reported 13 patients with sebaceous carcinoma arising in the eyelid who were treated with radiotherapy [11]. In that study, the radiation dose ranged from 50 to 66.6 Gy and the 5-year local progression-free rate was 100%. Takagawa et al. reported 83 patients with localized sebaceous carcinoma of the eyelid treated with radiotherapy, and the outcome was much worse than that reported by Hata et al.; the radiation dose ranged from 48 Gy to 70.4 Gy, and the 7-year local control rate was 52.3% [11,12]. These studies showed that the dose required for tumor control remains unclear; nevertheless, these data may indicate that 60-66 Gy, which is used to eradicate common skin cancers including basal cell carcinoma and cutaneous squamous cell carcinoma, may be insufficient to control sebaceous carcinoma [13].

Sebaceous carcinoma that arises from the extraocular location is even rarer, and only one report of radiotherapy aside from adjuvant therapy and salvage therapy was known. Oshiro et al. reported a case of sebaceous carcinoma arising from the tongue [14]. Radiotherapy was performed with 63 Gy in 35 fractions in addition to concurrent superselective intra-arterial chemotherapy because the patient refused surgery. Complete response of the primary lesion after radiotherapy was confirmed through multiple biopsies. The patient expired 17 months after completing the initial treatment by distant metastases. In the current case, the patient was at an extremely old age and was thus considered ineligible for surgery and chemotherapy.

Although Oshiro et al. concluded in their report that radiotherapy combined with superselective intra-arterial chemotherapy can be an effective treatment for sebaceous carcinoma of the tongue, the optimal radiation dose for controlling extraocular sebaceous carcinoma remains unclear [14]. At this stage, extraocular sebaceous carcinoma harbors biological characteristics or a radiosensitivity that is different from those of sebaceous carcinoma of the eyelid. Taking all of these factors into consideration, using H_2_O_2_-soaked gauze in combination with 60-Gy radiotherapy was tried to be used with the aim of potential radiosensitization. As a result, combined radiotherapy plus H_2_O_2_-soaked gauze resulted in complete tumor remission with tolerable dermatitis, indicating that this treatment can be a viable option for inoperable periauricular sebaceous carcinoma.

The putative mechanisms of the radiosensitizing effect of H_2_O_2_ are based on reoxygenation of the radioresistant hypoxic tumor microenvironment, the indirect effect of inducing the intracellular production of reactive oxygen species to increase the induction of DNA double-strand breaks, and the influence of several radioresistance-associated signaling pathways [7,15-17]. Ogawa et al. first described the radiosensitization strategy using an H_2_O_2_-soaked gauze in 2008 [6]. In that study, the authors reported five cases of locally advanced and/or recurrent tumors exposed to the skin surface, which were treated with electrons plus an H_2_O_2_-soaked gauze. More recently, Oike et al. reported a case of metastatic breast cancer with palliative radiotherapy with 51 Gy in 17 fractions using the H_2_O_2_-soaked gauze [7]. The tumor showed macroscopic complete remission three months after treatment, with the resolution of the patient’s symptoms, including bleeding and exudation. The patient experienced grade one radiation dermatitis, which resolved after radiotherapy without topical medication. Similarly, Shiba et al. reported a case of locally advanced breast cancer involving the skin surface that was treated with radiotherapy with 51 Gy in 17 fractions using the H_2_O_2_-soaked gauze, with favorable local effects observed [8]. The patient experienced grade three acute radiation dermatitis, which was treated with topical steroids and improved two months after radiation therapy. The tumor showed a complete clinical response to the primary tumor four months after radiotherapy. Together with the present case, these reports suggest the efficacy and tolerability of the treatment with electrons with the H_2_O_2_-soaked gauze. Nevertheless, solid evidence of the tolerable and optimal dose fractionation for this treatment is lacking, thus warranting further studies in a prospective setting for various cancers.

## Conclusions

The current study reported the first case of inoperable periauricular sebaceous carcinoma treated with definitive radiotherapy plus an H_2_O_2_-soaked gauze. Excellent local control was achieved at least eight months after treatment without severe adverse effects. In addition, the current case indicates that radiotherapy with an H_2_O_2_-soaked gauze can be an effective and tolerable treatment in inoperable patients with extraocular sebaceous carcinoma.
